# Plasmonic Light Scattering in Textured Silicon Solar Cells with Indium Nanoparticles from Normal to Non-Normal Light Incidence

**DOI:** 10.3390/ma10070737

**Published:** 2017-07-01

**Authors:** Wen-Jeng Ho, Jian-Cheng Lin, Jheng-Jie Liu, Chien-Wu Yeh, Hong-Jhang Syu, Ching-Fuh Lin

**Affiliations:** 1 Department of Electro-Optical Engineering, National Taipei University of Technology, No. 1, Section 3, Zhongxial East Road, Taipei 10608, Taiwan; t104658023@ntut.edu.tw (J.-C.L.); jjliu@mail.ntut.edu.tw (J.-J.L.); t103658018@ntut.edu.tw (C.-W.Y.); 2 Graduate Institute of Photonics and Optoelectronics, National Taiwan University, No. 1, Section 4, Roosevelt Road, Taipei 10617, Taiwan; f98941054@ntu.edu.tw (H.-J.S.); lincf@ntu.edu.tw (C.-F.L.)

**Keywords:** plasmonic light scattering, light trapping modes, indium nanoparticles (In NPs), textured silicon solar cells

## Abstract

In this study, we sought to improve the light trapping of textured silicon solar cells using the plasmonic light scattering of indium nanoparticles (In NPs) of various dimensions. The light trapping modes of textured-silicon surfaces with and without In NPs were investigated at an angle of incidence (AOI) ranging from 0° to 75°. The optical reflectance, external quantum efficiency (EQE), and photovoltaic performance were first characterized under an AOI of 0°. We then compared the EQE and photovoltaic current density-voltage (J-V) as a function of AOI in textured silicon solar cells with and without In NPs. We observed a reduction in optical reflectance and an increase in EQE when the cells textured with pyramidal structures were coated with In NPs. We also observed an impressive increase in the average weighted external quantum efficiency (∆EQE_w_) and short-circuit current-density (∆J_sc_) in cells with In NPs when illuminated under a higher AOI. The ∆EQE_w_ values of cells with In NPs were 0.37% higher than those without In NPs under an AOI of 0°, and 3.48% higher under an AOI of 75°. The ∆J_sc_ values of cells with In NPs were 0.50% higher than those without In NPs under an AOI of 0°, and 4.57% higher under an AOI of 75°. The application of In NPs clearly improved the light trapping effects. This can be attributed to the effects of plasmonic light-scattering over the entire wavelength range as well as an expanded angle of incident light.

## 1. Introduction

Solar cells based on silicon wafers account for a major share of the photovoltaic market, due to the abundance of the constituent materials and the maturity of the underlying technologies [1]. Pyramidal textures and antireflective coatings are commonly used to enhance the efficiency of these devices [2,3,4,5]. Unfortunately, randomly spaced upright pyramids trap only half of the light that returns to the front surface, even after two passes through the substrate. Scientists have recently discovered that the plasmonic effects induced by metallic nanoparticles can greatly enhance the performance of solar cells [6,7]. The integration of plasmonic nanoparticles into photovoltaic devices can greatly improve the light-trapping, absorption, and short-circuit photocurrent density of organic solar cells, dye-sensitized solar cells, and textured silicon solar cells [8,9,10,11,12,13,14,15]. The combination of geometric features with plasmonic metallic nanoparticles (NPs) has also been shown to trap more light, while enabling a wider angle of incident light [11,12,16,17,18]. Nonetheless, it is important to further elucidate the simultaneous interaction of light with metal NPs and textured silicon surfaces, in order to maximize the transfer of power generating electrons.

The plasmonic resonance of indium (In) NPs occurs in the ultraviolet range [19,20], where the intensity of solar irradiance is relatively low (negligible). Indium NPs have demonstrated considerable potential in preventing the Fano effect (i.e., destructive interference between scattered and un-scattered lights), which can greatly reduce light absorption in solar cells [21]. In this study, we proposed light-trapping models at incident angles of 0°, 35.3°, and 54.7° and investigated the performance enhancement in textured silicon solar cell coated with plasmonic In NPs of various dimensions with incident angle of light from 0° to 75° [11,22,23]. Optical reflectance, external quantum efficiency (EQE), and photovoltaic performance were shown to depend on the dimensions of the In NPs as well as the incident angle of light. EQE is the ratio of the number of charge carriers collected by the solar cell to the number of photons of a given energy striking the solar cell from outside. The light-trapping performance of cells with In NPs is superior to that of cells without In NPs, due to the effects of plasmonic light-scattering over the entire wavelength range while enabling a wider angle of incident light.

## 2. Experiments and Light-Trapping Modes

### 2.1. Experiments

#### 2.1.1. Bare Textured C-Si Solar Cells: Fabrication and Characterization

**Figure 1:**
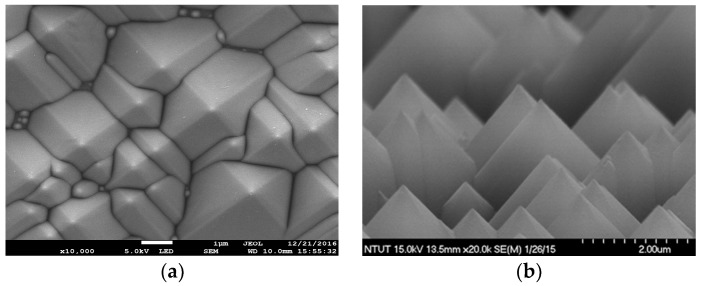
(**a**) Top-view and (**b**) side-view SEM images of etched surface pyramidal-structure.

In this study, Czochralski (CZ)-grown, (100)-oriented, boron-doped C-Si wafers (180 μm-thick) with a resistivity of 10 Ω·cm were first cleaned using the standard Radio Corporation of America (RCA) cleaning process. The surface of the C-Si substrate was etched using an anisotropic solution of H_2_O/KOH/IPA at 80 °C for 20 min to produce a surface texture comprising a random arrangement of pyramidal structures. Figure 1a,b and present top-view and side-view scanning electron microscope (SEM) images showing the etched surface. The minimum and maximum spacing between pyramids were respectively 4 μm and 7 μm, whereas the minimum and maximum heights were 4 μm and 7 μm. A 0.3 μm-thick n^+^-Si emitter layer with a sheet resistance of approximately 80 Ω/sq was applied to the textured C-Si substrates using a POCl_3_ diffusion process in a tube diffusion chamber at 850 °C for 3 min. The phosphorous silicate glass remaining on the surface was removed using a buffered oxide etchant (BOE) prior to the deposition of a 70-nm-thick silicon nitride antireflective film using plasma-enhanced chemical vapor deposition. A film of aluminum (Al) was deposited to a thickness of 600 nm on the rear surface using electron-beam (E-beam) evaporation and then annealed in a rapid thermal annealing (RTA) chamber at 450 °C for 5 min under ambient N_2_/H_2_ to form a back electrode. Finally, top contact grid-electrodes comprising a 600-nm Ag film was fabricated using lift-off photolithography and E-beam evaporation to produce bare textured C-Si solar cells (reference cells). The optical reflectance, external quantum efficiency (EQE), and photovoltaic performance of bare textured silicon solar cells were characterized as a reference for subsequent comparisons.

#### 2.1.2. Plasmonic Textured C-Si Solar Cell Fabrication and Characterization

**Figure 2:**
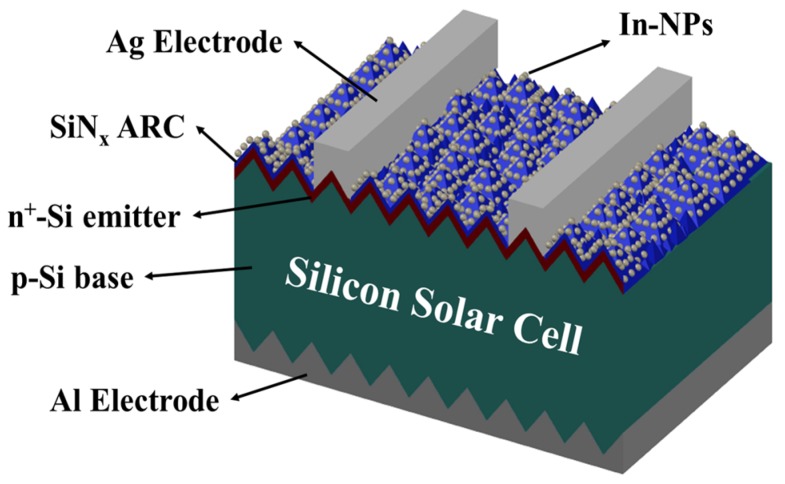
Schematic diagram showing textured C-Si solar cell coated with layer of In NPs.

Indium film was respectively deposited on textured C-Si solar cells to a thickness of 3.8, 5, and 6 nm. The samples then underwent annealing in an RTA chamber at 200 °C for 20 min under ambient H_2_ to transform the indium film into In NPs. Figure 2 presents a schematic diagram of a textured C-Si solar cell coated with a layer of In NPs. We also produced a textured C-Si solar cell without any In NPs for comparison. The resulting textured surfaces were examined using SEM (Hitachi S-4700, Hitachi High-Tech Fielding Corporation, Tokyo, Japan). The optical reflectance of the solar cells was characterized using a UV/VIS/NIR spectrophotometer (PerkinElmer LAMBDA 35, Waltham, MA, USA). The external quantum efficiency (EQE) of the cells was also measured over a range of wavelengths from 350 nm to 1100 nm, using a solar cell spectral response measurement system (EQE-RQE-R3015, Enli Technology Co., Ltd., Kaohsiung, Taiwan). The photovoltaic current density-voltage (J-V) characteristics of solar cells with and without In NPs were measured using a solar simulator (XES-151S, San-Ei Electric Co., Ltd., Osaka, Japan) and source meter (Keithley 2400, Keithley Instruments, Inc., Solon, OH, USA) at 25°C. The solar simulator was calibrated according to an NREL-certified crystalline silicon reference cell (PVM-894, PV Measurements Inc., Boulder, CO, USA) before obtaining measurements.

### 2.2. Light-Trapping Modes

Surface texturing is commonly applied to wafer-based silicon solar cells to reduce optical reflection. This often involves the anisotropic etching of a (100)-oriented silicon substrate into pyramidal structure. The size, height, and base width of the pyramids are usually on the order of several micrometers. The micro-pyramids redirect light to produce multiple reflections in order to increase the likelihood of light absorption by the silicon substrate. We also examined the influence of the angle of incidence on the reflection of light rays from {111} facets. These results led to the formulation of models pertaining to the periodic pyramidal structure with and without In NPs for use in deriving the total reflection due to the contribution of primary and second reflections.

#### 2.2.1. Light-Trapping Modes of Textured Surface

**Figure 3:**
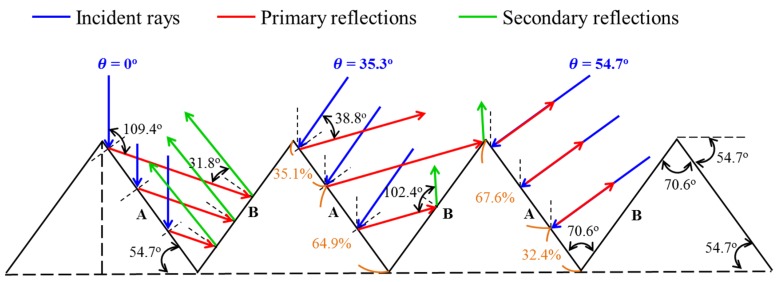
Schematic diagram showing the periodic pyramidal-structure.

Figure 3 presents a two-dimensional (2D) schematic model of a periodic pyramidal structure. The angle of incidence was gradually increased from θ = 0° to 35.3° until the incident light rays (blue color) were tangential to facet B in order to determine whether the angle of incidence (prior to the primary reflections (red color) from surface A) could produce secondary reflections (green color) on surface B. At angles higher than 35.3°, the reflection from facet B vanished until θ = 54.7°, at which point the incident rays were normal to facet A. At incident angles exceeding 54.7°, the reflected rays were directed away from the substrate, which means that no secondary reflection would occur at these angles. We assumed that the primary reflection coefficient from facets A and B was *r_A_* and *r_B_*, respectively. The primary reflection coefficient is given as follows:

(1) $$\left|r_{A}\right|=\left|r_{B}\right|=\frac{\cos(\theta )-\sqrt{{\left(\frac{n_{Si}}{n_{air}}\right)}^{2}-\sin^{2}(\theta )}}{\cos(\theta )+\sqrt{{\left(\frac{n_{Si}}{n_{air}}\right)}^{2}-\sin^{2}(\theta )}}$$

We derived the total reflectance (*R_T_*) by estimating the fraction of light rays incidental to each facet as well as their contributions to the total reflectance. Thus, for θ = 0°, the total reflectance is given as follows:

*R_T-0_*_°_ = *r_A_* × *r_B_* (2)

For θ = 35.3°, the total reflectance is

*R_T-35.3°_* = 0.351*r_A_* + 0.649*r_A_* × *r_B_* (3)

For θ = 54.7°, the total reflectance is

*R_T-54.7°_* = 0.676*r_A_* (4)

Thus, we obtained *R_T-54.7°_* > *R_T-35.3°_* > *R_T-0°_* when the angle of incidence was set from 0° to 35.3° to 54.7°.

#### 2.2.2. Light-Trapping Mode of a Textured Surface with In NPs

**Figure 4:**
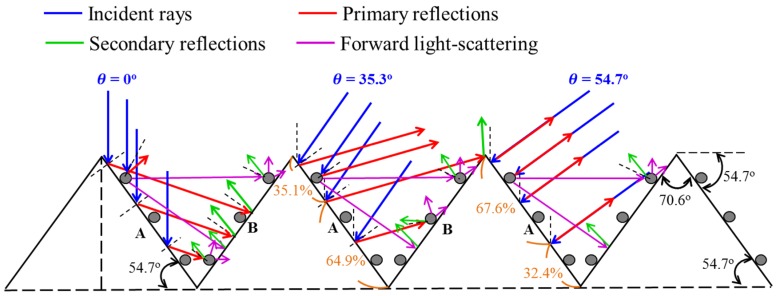
Schematic diagram showing periodic pyramidal-structure coated with In NPs.

Figure 4 presents a two-dimensional (2D) schematic model of a periodic pyramidal structure with In NPs. The coverage of the In NPs over the textured surface was approximately 30%. The reflection coefficient on facets A and B was *r_NPs_*_-A_, *_rNPs_*_-B_, which is greater than *r_A_* or *r_B_*. For the sake of simplification, we assumed that the light rays striking the In NPs would generate an equal amount of backward light-scattering (50%) and forward light-scattering (50%). Reflections from the micro-pyramids and In NPs redirected and scattered the light to produce multiple reflections with the aim of increasing the probability of light being absorbed by the silicon substrate. Thus, for θ = 0°, the total reflectance is given as follows:

*R_T-0°-NPs_* = 0.7*r_A_* {0.7*r_B_* + 0.3 *r_NPs-B_* [0.5 (0.7*r_A_* + 0.3*r_NPs-A_*) + 0.5*r_B_*]} + 0.3*r_NPs-A_* [0.7*r_B_* + 0.3 (0.5*r_NPs-B_* + 0.5*r_B_*)] (5)

In the case of θ = 35.3°, the total reflectance is obtained as follows:

*R_T-35.3°-NPs_* = 0.7 {0.351*r_A_* + 0.649*r_A_* [0.7*r_B_* + 0.3 (0.5*r_NPs-B_* + 0.5*r_B_*)]} + 0.3 *r_NPs-A_* {(0.5 × 0.351) *+* (0.5 × 0.649) × [0.7*r_B_* + 0.3 (0.5*r_NPs-B_* + 0.5*r_B_*)]} (6)

For θ = 54.7°, the total reflectance is obtained as follows:

*R_T-54.7°_*_-*NPs*_ = 0.7(*r_A_* × 0.676*) +* 0.3*r_NPs-A_* {(0.5 × 0.676) + (0.5 × 0.676)[0.7*r_B_* + 0.3(0.5*r_NPs-B_* + 0.5*r_B_*)]} (7)

Based on Equations (2)–(7), the total reflectance of the textured surfaces with In NPs was as follows: *R_T-54.7°-NPs_* > *R_T-35.3°-NPs_* > *R_T-0°-NPs_*. This can be used to obtain the total reflectance, as follows*: R_T-54.7°_* > *R_T-54.7°-NPs_*, *R_T-35.3°_* > *R_T-35.3°-NPs_*, *R_T-0°_ > R_T-0°-NPs_*. We can see that the total reflectance of the textured silicon solar cells with In NPs is less than that of the textured cell without In NPs at incident angles from 0° to 54.7°.

## 3. Results and Discussion

**Figure 5:**
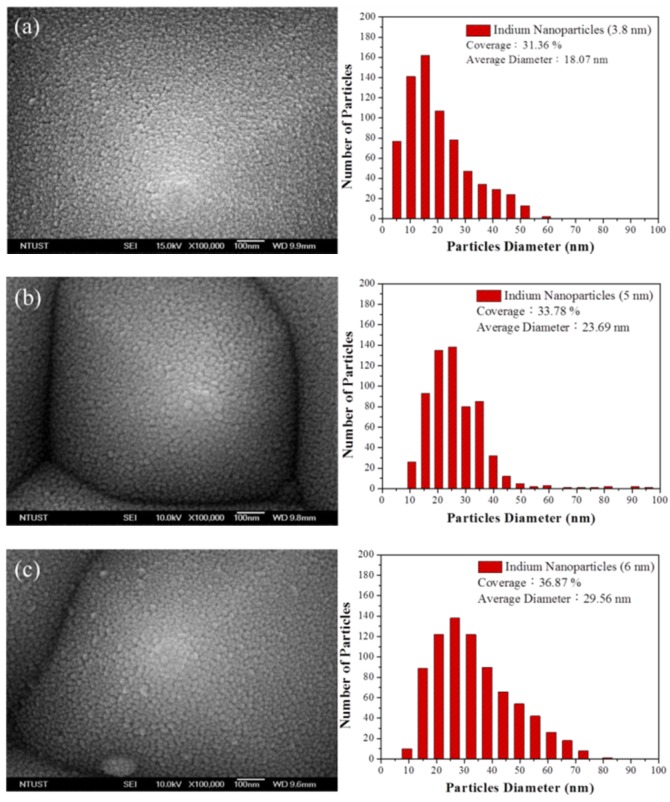
Top-view SEM images and particles profile of (**a**) 3.8 nm; (**b**) 5 nm; (**c**) 6 nm thick indium film deposited and annealed on the pyramidal surface structure of plasmonic textured C-Si solar cells.

**Figure 6:**
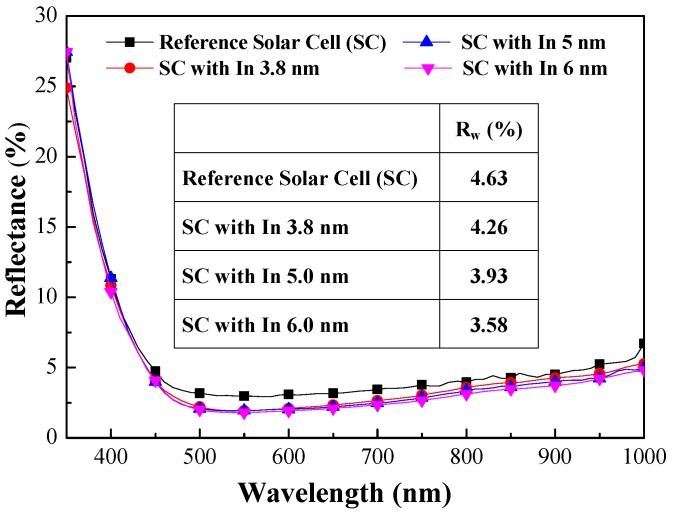
Optical reflectance of reference silicon solar cell (textured but without NPs) and textured cells coated with In NPs of various dimensions.

Figure 5 presents a top-view SEM image and particle profile of In NPs on the textured surface of textured surface of a C-Si solar cell. The size distribution and the coverage of the In NPs were calculated using Image-J software (National Institutes of Health, Bethesda, MD, USA). We compared the averaged dimensions and coverage of the In NPs according to the thickness of the In layer prior to annealing, as follows: (a) 3.8 nm thick (18.07 nm) (31.36%), (b) 5 nm thick (23.69 nm) (33.78%), and (c) 6 nm thick (29.65 nm) (36.87%). Clearly, a thicker film of In resulted in NPs with larger dimensions and greater coverage. Figure 6 presents the optical reflectance of the reference silicon solar cell (textured but without NPs) and solar cells coated with NPs of various dimensions. The reflectance values of the cells with In NPs were lower than those of the reference cell without In NPs, due to plasmonic forward light-scattering afforded by the NPs. The reflectance shown in Figure 6 was measured on the sample with metallic grid-electrodes which also provide some other reflection. Therefore, the fabricated sample has a reflectance minimum around 2.5%. Solar cells with lower reflectance are able to trap more incident light. In this study, we observed that the reflectance of cells with larger In NPs was slightly lower than that of cells with smaller particles. The larger particles also produced a slight red shift in the reflectance curves. For the sake of clarity, we calculated the average weighted reflectance (*R_W_*) of the cells as follows:

(8) $$R_{W}=\frac{\int_{350\;nm}^{1000\;nm}R(\lambda )\varphi_{\mathrm{ph}}(\lambda )d\lambda }{\int_{350\;nm}^{1000\;nm}\varphi_{ph}(\lambda )d\lambda }\times 100\%$$

where *R(λ)* is the reflectance at a given wavelength (*λ*) and *φ(λ)* is the photon flux of AM 1.5 G at that wavelength (*λ*). The *R_W_* values are summarized in the inset of Figure 6. The *R_W_* values of the cells with In NPs were lower than those of the reference cells, which indicates that they are able to trap more of the incident light.

**Figure 7:**
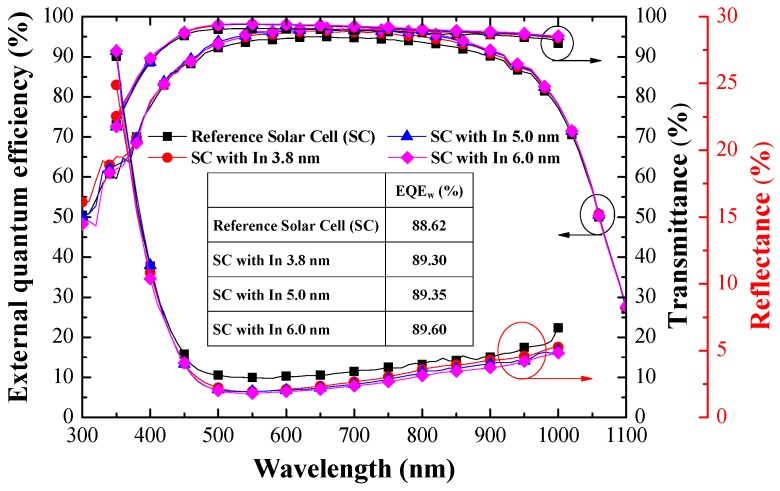
EQE response of reference solar cell (textured silicon but without NPs) and textured silicon solar cell coated with In NPs of various dimensions under normal incidence (θ = 0°).

Figure 7 presents the EQE response of the reference silicon solar cell (textured but without NPs) and textured cells coated with In NPs of various dimensions. The EQE values of cells with In NPs were higher than that of the reference solar cell across the entire wavelength range, due to the effects of plasmonic forward scattering induced by In NPs. A higher EQE value means that more incident light is absorbed (trapped) within the solar cell. The EQE values of cells with In NPs of larger dimensions were slightly higher than those of cells with smaller particles. For the sake of clarity, we calculated the average weighted EQE (*EQE_W_*) of the cells as follows:

(9) $$EQE_{W}=\frac{\int_{300\;nm}^{1100\;nm}EQE(\lambda )\varphi_{ph}(\lambda )d\lambda }{\int_{300\;nm}^{1100\;nm}\varphi_{ph}(\lambda )d\lambda }\times 100\%$$

where *EQE(λ)* is the EQE response at a given wavelength (*λ*). The *EQE_W_* values are summarized in the inset of Figure 7. Overall, the EQE response values are in good agreement with the optical reflectance results. Figure 7 also plots the reflectance and transmittance values to enable a comparison of light-trapping performance. The absorption of incident photons within the active silicon layer depends on the optical transmittance (*T*): *T (λ)* = 1 − *R (λ)* − *A (λ)*, where *R* is the total reflectance and *A* is the total absorption (disregarded). This means that the EQE values increase with an increase in the optical transmittance and/or with a decrease in optical reflectance. In this work, the EQE response results appear to be in good agreement with the proposed light trapping modes of the textured silicon solar cells with and without In NPs under normal incidence (θ = 0°).

**Figure 8:**
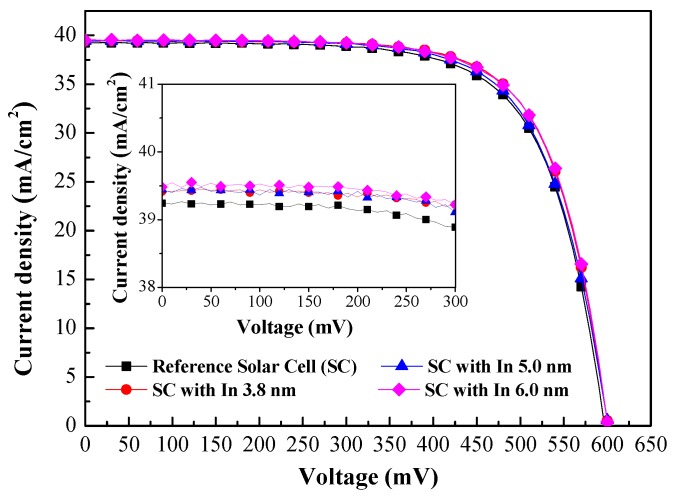
Photovoltaic J-V curves of reference solar cell (textured silicon but without NPs) and textured silicon solar cell coated with In NPs of various dimensions under normal incidence (θ = 0°).

Figure 8 presents the photovoltaic J**-**V curves of the reference solar cell (textured silicon but without NPs) and textured silicon solar cell coated with In NPs of various dimensions under normal incidence (θ = 0°). Table 1 lists the photovoltaic performance of the textured silicon solar cells evaluated in this work. We compared the short-circuit current-density (J_SC_) values and conversion efficiency (η) according to the thickness of the In layer prior to annealing, as follows: (a) 3.8 nm thick (39.41 mA/cm^2^) (16.63%), (b) 5 nm thick (39.44 mA/cm^2^) (16.71%), and (c) 6 nm thick (39.48 mA/cm^2^) (16.76%). Compare these values to those from the cell without In NPs (JSC = 39.24 mA/cm^2^ and η = 16.30%), this is a clear indication that the increase in JSC can be attributed to the light trapping effects of plasmonic forward light-scattering induced by the In NPs. We also observed that the effects are more pronounced in samples with In NPs of larger dimensions when the incident angle was 0°. These values are in agreement with the EQE response values. 

**Figure 9:**
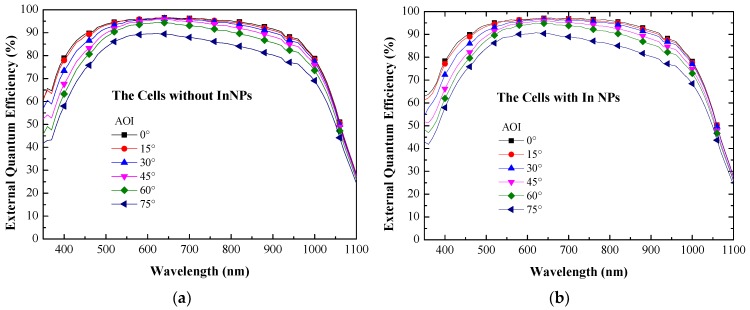
EQE response of (**a**) reference silicon solar cell (textured but without NPs) and (**b**) textured silicon solar cell coated with In NPs, measured at AOI of 0°–75°.

**Figure 10:**
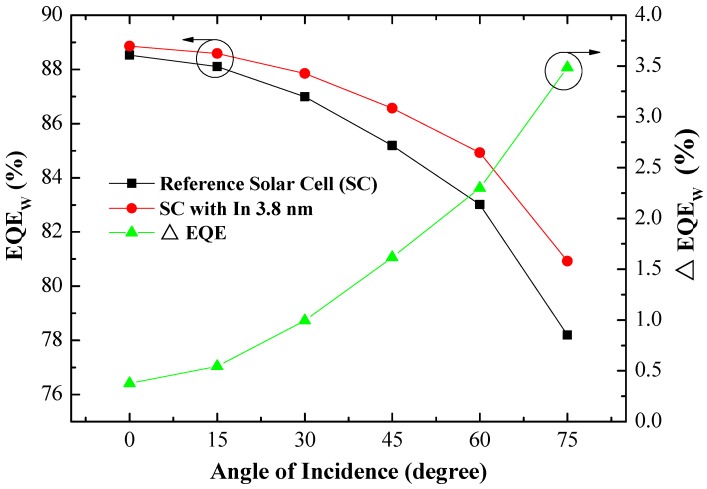
Average weighted EQE (*EQE_W_*) of cells at each AOI, as calculated using Equation (9).

Figure 9 presents the EQE response of (a) the reference solar cell (textured silicon but without NPs) and (b) textured silicon solar cell coated with In NPs of various dimensions measured under incident angles of 0°–75°. Increasing the incident angle led to a gradual decrease in the EQE response values of cells with and without In NPs across the entire range of wavelengths. The measured EQE results are consistent with the proposed light-trapping modes; i.e., the total reflectance in both modes should increase with an increase in the incident angle. When the incident angle was increased to beyond 60°, the EQE values presented a significant decrease at wavelengths of 600–1000 nm. We also calculated the average weighted EQE (*EQE_W_*) and *EQE_W_* improvement (∆*EQE_W_*) at each angle of incidence (AOI) using Equation (9), the results of which are presented in Figure 10 and summarized in Table 2. The *EQE_W_* results demonstrate that textured silicon solar cells coated with a layer of In NPs achieve higher *EQE_W_* values, regardless of the angle of incidence. They also exhibited a far wider angle of acceptance in achieving a given *EQE_W_* value. In general, a high *EQE_W_* value results in solar cells with high J_SC_ and thus higher efficiency. The application of In NPs also improved ∆*EQE_W_* (3.48%) at an incidence angle of 75°, compared to cells without a layer of In NPs.

**Figure 11:**
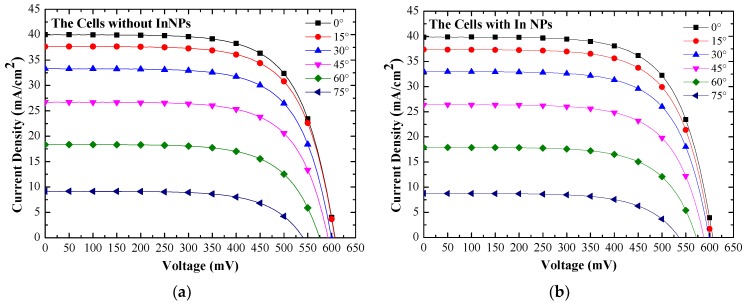
Photovoltaic J-V curves of (**a**) reference silicon solar cell (textured but without NPs) and (**b**) textured silicon solar cell coated with In NPs measured under an AOI of 0°–75°.

**Figure 12:**
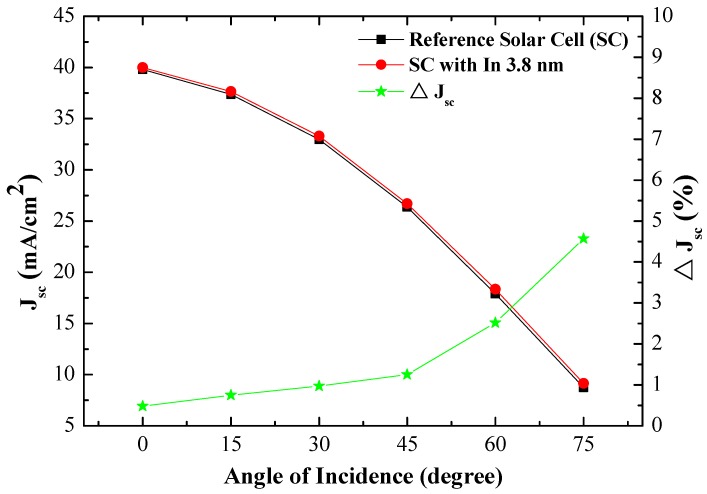
Improvements in J_SC_ and J_SC_ as a function of incident angle in textured silicon solar cells with and without a layer of In NPs.

Figure 11 presents the photovoltaic J-V curves of (a) reference silicon solar cell (textured but without NPs) and (b) textured cell coated with a layer of In NPs, as measured under AOI of 0°–75°. Table 3 lists the photovoltaic performances of the textured silicon solar cells evaluated in this work under AOI of 0°–75°. An increase in AOI from 0° to 75° led to a gradual decrease in J_SC_ and open-circuit voltage (V_OC_). J_SC_ and V_OC_ are proportional to the EQE value. Thus, the η of cells with NPs would be higher than that of the cells without NPs at incident angles from 0° to 75°. Figure 12 plots the improvements in J_SC_ and J_SC_ as a function of incidence angle. The trend seen in J_SC_ improvement is in agreement with the improvement in *EQE_W_* at incident angles of 0°–75°.

## 4. Conclusions

In this study, we conducted a comprehensive investigation into the light-trapping performance of textured silicon solar cells with and without In NPs at incidence angles from 0° to 75°. We also proposed light-trapping modes on textured surfaces with and without In NPs for light from normal to non-normal angles of incidence. We then compared the *EQE* and photovoltaic current density-voltage (*J-V*) of all solar cells with and without In NPs as a function of the angle of incidence. We observed a reduction in optical reflectance and increase in *EQE* following the application of In NPs over the pyramidal surface structure format incidence angles from 0° to 75°. This greatly improved the light-trapping performance of the solar cells, due to the effects of plasmonic forward light-scattering.

## Figures and Tables

**Table 1:** Photovoltaic performances of textured silicon solar cells evaluated in this work.

	J_sc_ (mA/cm^2^)	V_oc_ (mV)	η (%)	△J_sc_	△η
Ref. Solar Cell	39.24	596.74	16.30	--	--
SC with In 3.8 nm	39.41	600.89	16.63	0.43%	2.02%
SC with In 5.0 nm	39.44	600.06	16.71	0.51%	2.51%
SC with In 6.0 nm	39.48	600.57	16.76	0.61%	2.81%

**Table 2:** Average weighted EQE (*EQE_W_*) and improvement in *EQE_W_* (∆*EQE_W_*) at each AOI.

	0°	15°	30°	45°	60°	75°
*EQE_W_* (%)	88.52	88.1	86.98	85.18	83.01	78.19
*EQE_W-In NPs_* (%)	88.85	88.58	87.85	86.56	84.92	80.92
*△EQE_W_* (%)	0.37	0.54	0.99	1.61	2.29	3.48

**Table 3:** Photovoltaic performance of textured silicon solar cells evaluated in this work, as measured under AOI of 0°–75°.

	Reference Solar Cell			The Cell with In NPs		
**Angle**	**J_sc_ (mA/cm^2^)**	**V_oc_ (mV)**	**η (%)**	**J_sc_ (mA/cm^2^)**	**V_oc_ (mV)**	**η (%)**
0°	39.81	606.73	16.41	40.01	606.98	16.51
15°	37.36	602.8	15.3	37.64	606.35	15.64
30°	32.98	599.02	13.39	33.3	599.44	13.61
45°	26.37	588.01	10.44	26.7	592.33	10.72
60°	17.9	571.96	6.8	18.35	574.37	7.01
75°	8.75	535.66	3.02	9.15	540.31	3.21
